# A Bias‐Corrected Three‐Step Approach to Study the Association Between Aging Trajectories and Sociodemographic and Clinical Factors

**DOI:** 10.1002/alz.090926

**Published:** 2025-01-09

**Authors:** Weiyi Xia, Anum Zafar, Olga F Jarrín Montaner, Haiqun Lin

**Affiliations:** ^1^ Rutgers, The State University of New Jersey, Piscataway, NJ USA; ^2^ Rutgers, The State University of New Jersey, New Brunswick, NJ USA; ^3^ Rutgers, The State University of New Jersey, Newark, NJ USA

## Abstract

**Background:**

This work focuses on methodological aspects in assessing the association between group‐based aging trajectories and sociodemographic and clinical factors. The group‐based trajectories are derived from the latent class analysis of longitudinal variables of clinical interest. Each individual would be assigned a class membership with the highest posterior probability (modal assignment) and each class represents a particular pattern in the trajectory of the longitudinal variables. The Bolck–Croon–Hagenaars (BCH) method for bias adjustment in assessing the association of the latent class membership and external variables has increasingly become popular during the last twenty years.

**Method:**

The BCH method is the three‐step approach that adjusts for the misclassification error in the latent class assignment. In the first step, we fit a group‐based trajectory model. In the second step, we calculate the posterior probability of class membership of each individual which is assigned to the class with either modal assignment or proportional assignment to each of the classes. The BCH class assignment weights are estimated in this step as well. In the third step, association parameters between latent class and external variables are estimated using weights from the step two. We generate simulation data to evaluate the performance of the three‐step approach without BCH adjustment and the bias‐corrected BCH approach. Subsequently, the association analyses between aging trajectory and external variables of interest among Medicare beneficiaries were conducted.

**Result:**

The group‐based trajectory algorithm works well with truncated normal and ordered categorical longitudinal measurements among Medicare beneficiaries. We compute the inverse of the matrix and reweighting the data to obtain the desired joint probabilities of the latent class variables and external variables. The simulation result reveals the smallest bias when utilizing BCH‐proportional estimator and largest bias with the naive three‐step estimator. The BCH‐adjusted estimator outperforms the naive three‐step method in correcting bias.

**Conclusion:**

Our results show the feasibility of applying BCH in the group‐trajectory analysis for both continuous and categorical longitudinal indicators for latent classes. The bias‐corrected three‐step approach with BCH can effectively reduce the bias from the latent class misclassification error.

